# Immunotherapy Monitoring with Immune Checkpoint Inhibitors Based on [^18^F]FDG PET/CT in Metastatic Melanomas and Lung Cancer

**DOI:** 10.3390/jcm10215160

**Published:** 2021-11-03

**Authors:** Egesta Lopci

**Affiliations:** Nuclear Medicine Unit, IRCCS—Humanitas Research Hospital, Via Manzoni 56, 20089 Rozzano, MI, Italy; egesta.lopci@gmail.com or egesta.lopci@cancercenter.humanitas.it

**Keywords:** immunotherapy, checkpoint inhibitors, metabolic response, tumor response, [^18^F]FDG PET/CT, immuno-PET

## Abstract

Immunotherapy with checkpoint inhibitors has prompted a major change not only in cancer treatment but also in medical imaging. In parallel with the implementation of new drugs modulating the immune system, new response criteria have been developed, aiming to overcome clinical drawbacks related to the new, unusual, patterns of response characterizing both solid tumors and lymphoma during the course of immunotherapy. The acknowledgement of pseudo-progression, hyper-progression, immune-dissociated response and so forth, has become mandatory for all imagers dealing with this clinical scenario. A long list of acronyms, i.e., irRC, iRECIST, irRECIST, imRECIST, PECRIT, PERCIMT, imPERCIST, iPERCIST, depicts the enormous effort made by radiology and nuclear medicine physicians in the last decade to optimize imaging parameters for better prediction of clinical benefit in immunotherapy regimens. Quite frequently, a combination of clinical-laboratory data with imaging findings has been tested, proving the ability to stratify patients into various risk groups. The next steps necessarily require a large scale validation of the most robust criteria, as well as the clinical implementation of immune-targeting tracers for immuno-PET or the exploitation of radiomics and artificial intelligence as complementary tools during the course of immunotherapy administration. For the present review article, a summary of PET/CT role for immunotherapy monitoring will be provided. By scrolling into various cancer types and applied response criteria, the reader will obtain necessary information for better understanding the potentials and limitations of the modality in the clinical setting.

## 1. Introduction

Starting with the first outstanding results of the anti-cytotoxic T lymphocyte antigen-4 (CTLA-4) antibody, Ipilimumab, in melanoma [1] and following use of antibodies against programmed cell death protein 1 (PD-1) and its ligand PD-L1 (nivolumab, pembrolizumab, atezolizumab) in non-small cell lung cancer (NSCLC) [2,3,4,5], immunotherapy with checkpoint inhibitors has gradually changed the management of malignant tumors by improving the long term benefit and survival. Clinicians have become acquainted along the way with new ways of considering clinical benefit, meaning to recognize objective progression not necessarily as an upfront sign of treatment failure. From an imaging point of view, new semantic artifices have been implemented to help handle the variegated patterns of response that accompany treatment with immune checkpoint inhibitors (ICI). It is therefore not surprising that the number of response criteria has consequently increased, both for morphological and metabolic imaging (Table 1). For the present review article, a summary of the role of PET/CT for immunotherapy monitoring will be provided. By scrolling into various cancer types and applied response criteria, the reader will obtain necessary information for better understanding the potentials and limitations of the modality in the clinical setting.

## 2. New Concepts in Tumor Response during Immunotherapy

Born to overcome the limitations of conventional criteria, and driven by the need to avoid unnecessary treatment withdrawal, immunotherapy-derived response criteria have embraced concepts such as pseudo-progression, hyper-progression or dissociated progression to move beyond the immunotherapy era. Although previously described as an unconventional response pattern in gliomas treated with chemoradiotherapy [17], pseudo-progression is now more broadly associated with ICI and corresponds to the appearance of new lesions or the occurrence of tumor enlargement during therapy, followed by disease regression or stabilization at subsequent imaging [18]. The phenomenon is more frequent during anti-CTLA-4 therapy and tends to affect fewer cancer patients treated with anti-PD-1/L1 agents. Nevertheless, the rate of pseudo-progression in general does not exceed 10% [19,20].

Hyper-progression, on the other hand, refers to a very peculiar pattern of response to ICI, and was firstly described in 2016 by Champiat et al. [21]. Its occurrence ranges from 4% to 29%, proving a large variability of cases according to the casuistics [20,22]. Substantially, hyperprogressive disease (HPD) corresponds to a massive increase of tumor burden, over twice the amount compared to (prior to) treatment start. Notwithstanding, controversies exist on the exact way HPD is defined in clinical practice. While Champiat et al. [21] defined HPD as a twofold or greater increase of tumor growth rate (TGR) during immunotherapy [20], other authors used different descriptions. For instance, Kato et al. defined HPD as a time to treatment failure (TTF) < 2 months, a 50% increase in tumor burden compared to pre-immunotherapy imaging obtained within 2 months of the treatment initiation, and > 2-fold increase in progression pace [20,23]. In other cases, like for Saâda-Bouzid et al., HPD could be computed based on tumor growth kinetic ratio (TGKR), where TGK is defined as the difference of the sum of the largest diameters of target lesions per unit of time, which in the case of HPD has to be ≥ 2 when compared to baseline [20,24,25]. More simply, Matos et al. [26] used as parameter for HPD a 40% increase of the sum of the target lesions from baseline to the first evaluation and/or an increase of 20% plus the appearance of new lesions in two different organs [27]. Although comparison only to baseline imaging, without utilization of data before treatment start, has made some authors define as “fast progression” rather than “hyperprogression” the cases reported by later authors [24,25,26], strictly speaking the occurrence of this “non-response”, is in any of the cases, a dramatic failure. In fact, patients with this type of progression during ICI (call it “hyper-” or “fast”) have a worse outcome with a significantly shorter survival rate [20,21,22,23,24,25,26,28].

To add further confusion to the already intricate situation, recently a new pattern of tumor behavior during ICI has been described in advanced lung cancer [29,30]; this consists of a “dissociated response”, i.e., a contemporary shrinkage of some tumor lesions along with the increase of others in various organs [18], which occurs in around 10% of patients [31]. Given the potential benefit still obtainable for patients showing an immune dissociated response (iDR), some authors [30] have suggested iDR as a surrogate marker of favorable outcome and treatment efficiency [31].

**Figure 1:**
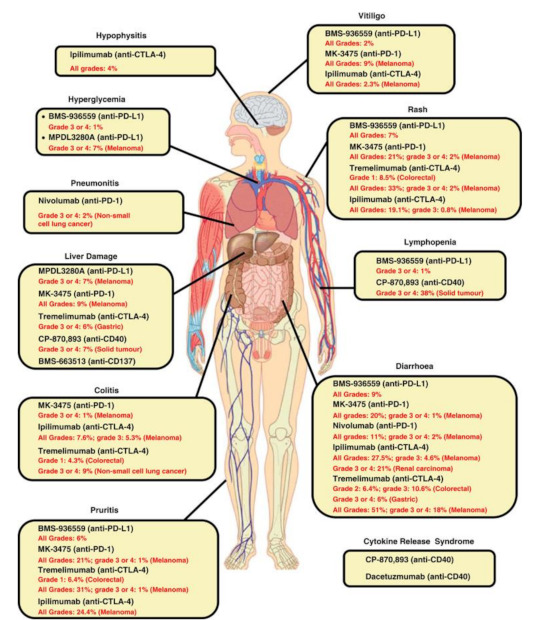
Spectrum of irAEs associated with immunomodulatory antibodies (available via license: Creative Commons Attribution-NonCommercial-NoDerivs 3.0 Unported, as published by Liu J, et al. Clin. Transl. Immunol. 2014, 3, e22) [35].

Along with the abovementioned new patterns of response, immunotherapy with ICI can determine several immunologically mediated alterations of healthy tissues and organs, also known as immune-related adverse events (irAEs) [18]. The incidence of these events is higher for anti-CTLA-4 antibodies (80%) and during combination therapy, while it reaches in general 27% for anti-PD-1 and 17% for anti-PD-L1 regimens [18,32]. The occurrence of irAEs, based on the severity of the event, might require immediate ICI discontinuation [33,34]. This will not necessarily prevent fatality, which is surprisingly related to colitis in 70% of the cases treated with anti-CTLA-4, followed by pneumonitis (35%), hepatitis (22%) and neurotoxicities (15%) for anti-PD-1/anti-PD-L1 antibodies [33,34]. From an imaging point of view, irAE interpretation can sometimes be as challenging as other unconventional patterns of response described during ICI. Given the potentially fatal events related to their occurrence, it is fundamental to be aware of their appearance and describe them promptly in the report and to the clinician treating the patient (Figure 1) [35]. Notwithstanding, there is also a positive aspect with irAEs, which is their potential predictive role for treatment benefit. Indeed, being an expression of immune system response, although abnormal and undesirable in most cases, irAEs represent a precognitive sign of longer progression-free (PFS) and overall survival (OS) [36]. From first reports to later meta-analyses, irAE development seems to be positively associated with overall response rate (ORR), PFS, and OS in patients treated with immunotherapy, regardless of lesion site, type of ICI and irAE [36,37], although, grade 3 or higher toxicities have resulted prognostically in worse OS [37].

## 3. Response Assessment in Solid Tumors Treated with Checkpoint Inhibitors

**Figure 2:**
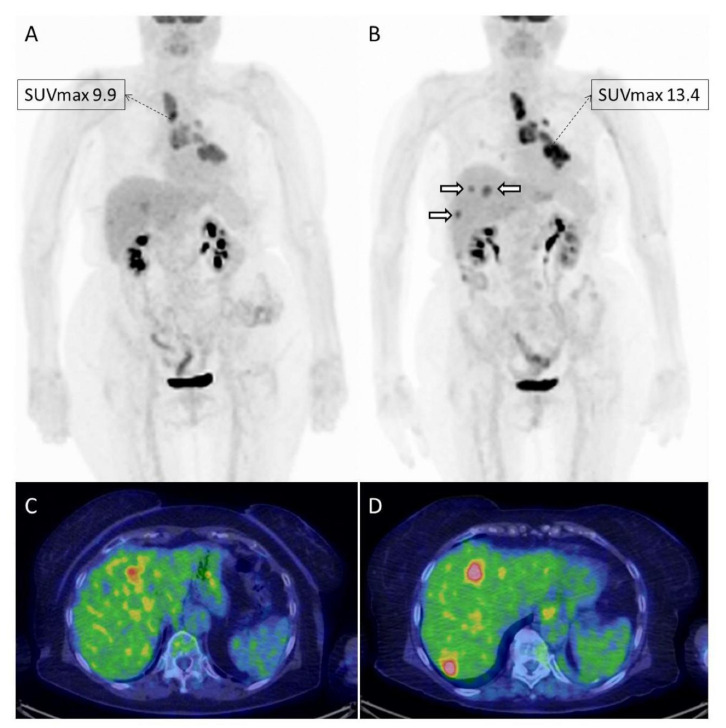
Example of a 78-year old female with advanced NSCLC treated with nivolumab and imaged with [^18^F]FDG PET/CT at baseline (**A**,**C**) and after 4 cycles of therapy (**B**,**D**). The patient resulted in overall stable on morphological imaging performed prior to PET/CT, which on the contrary documented a progressive metabolic disease. In fact, the tumor had an increase in metabolism (SUVmax and MTV), and showed the appearance of new lesions in the liver ((**B**); white hollowed arrows), only partially detectable on baseline imaging.

**Figure 3:**
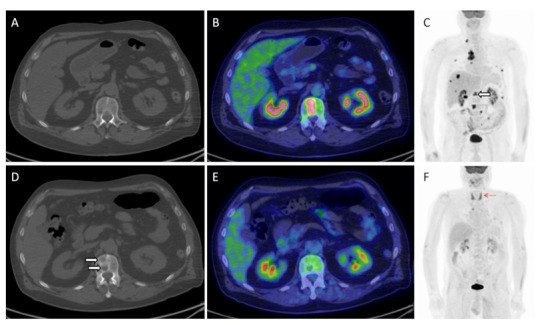
Herein, the imaging findings of a 66-year old male with metastatic NSCLC investigated before (**A**–**C**) and after 3 cycles of pembrolizumab (**D**–**F**). An overall response to treatment is easily visible on MIP (maximal intensity projection) images (**C**,**F**), including a complete metabolic remission of all bony lesions ((**C**); white hollowed arrow). On the contrary, morphological imaging proved the appearance of a new bone lesion in the first lumbar vertebra ((**A**,**D**); white arrows), which in fact corresponded to a healed metastasis on PET/CT (**B**,**E**). Note also the appearance of diffuse thyroid uptake ((**F**); red arrow), consistent with thyroiditis, one of the irAEs that typically predicts treatment response and good patient’s outcome.

Keeping in mind the abovementioned peculiarities of imagine interpretation during ICI, imagers require adequate instruments to assess immunotherapy benefit, which from a metabolic point of view consists mainly in the use [^18^F]FDG PET/CT for response assessment (Figure 2 and Figure 3). As previously anticipated, quite an extensive number of response criteria have been proposed for this purpose in recent years (Table 1). During initial studies, consolidated criteria, such as EORTC (European Organization for Research and Treatment of Cancer) [11] and PERCIST (PET Response Criteria in Solid Tumors) [12], have represented the simplest way to assess tumor response, followed later by subsequent adaptations to ICI. This is the case in the instance of PECRIT criteria (PET/CT Criteria for early prediction of Response to Immune checkpoint inhibitor Therapy), introduced by Cho et al. [16], which combine both morphologic (contemplating a change in the sum of diameters of target lesions according to RECIST 1.1) and metabolic response (i.e., a reduction in the SULpeak > 15.5% for the hottest lesion on PET) to assess clinical benefit of ICI. Other authors have introduced PERCIMT (PET Response Evaluation Criteria for IMmunoTherapy), firstly described in melanoma patients [13]. Herein, the appearance of up to four new lesions, depending on their size (Table 1), can be tolerated to obtain clinical benefit (CB) and support treatment continuation [13,38]. More recently, other alternative approaches to PERCIST have been used, including iPERCIST [15] and immunotherapy-modified PERCIST5 (imPERCIST) [14]. For the latter, the definition of a progressive metabolic disease (PMD) becomes less stringent, requiring in fact an increase in the sum of SULpeaks of 30%, with new lesions being eventually included in the sum of SULpeak [14,18]. The principle behind all these new adaptations is substantially the same: to avoid unnecessary and premature treatment withdrawal during immunotherapy. but can we depict one of them as the best response criteria for response assessment during ICI? Actually, not. Some reports have attempted to compare various methods, particularly in melanoma and NSCLC patients [14,38,39,40,41,42,43], proving the superiority of some of the utilized criteria over others (Table 2). Ultimately, all available response criteria, metabolic or morphological, retain the capability to predict response and outcome. What makes one criteria better than the other is most likely to be the interpretation ability of the imager and the correct contextualization of the results into clinical practice. This should not limit, in any case, the continuous research in the field, since robust data must be produced to optimize response criteria for response assessment during ICI, not forgetting the absolute necessity to ascertain the perfect timing for treatment discontinuation for patients to receive long-term clinical benefit.

## 4. Combined Parameters for Outcome Prediction

To date, special attention has been given to other parameters obtainable from [^18^F]FDG PET/CT during ICI. Not just standardized uptake value (SUV), but also metabolic tumor volume (MTV) and total lesion glycolysis (TLG), have been investigated at baseline and during treatment as absolute values or as variations to predict response and outcome [28,47,51,53,54,55,56,58,59,66,67,68,69,70,71,72,73,74,76,77,78,79,82,83,86,87,88]. While SUV appears to be inversely correlated to response to ICI [66,70,80] with higher SUV values being in some reports indicative of treatment benefit, on the other hand higher MTV and TLG values result in negative predictive factors for patient outcome during ICI (Table 2). Recently, a linear positive correlation between SUVmax and tumor mutational burden (TMB), which represents one of the prognostic markers of response to immunotherapy, has been reported (*p* < 0.001) [95]. These data are in line with previous findings reporting a paradoxically higher SUV in patients responding to ICI, particularly referring to NSCLC [66,70,96]. This evidence also reflects other observations showing a positive correlation between SUV and checkpoints (i.e., PD-L1 or PD-1) and the immune infiltrate [96,97,98,99,100] in lung and other cancer types.

Of special interest also is the risk stratification of patients based on volumetric parameters already obtained at baseline, with patients having a higher MTV and TLG being at higher risk of poor outcome or HPD compared to others [53,55,71,73,74,76,80]. In this context, to further improve the predictive role, a combination of metabolic tumor burden (MTV and TLG) with other clinical parameters has been performed. In particular, circulating inflammatory markers, such as neutrophyl-to-lymphocyte ratio (NLR) and its derived value (dNLR) have proved to better stratify patients undergoing immunotherapy with ICI into risk groups (i.e., higher values predicting poor outcome), both at baseline and after treatment start [71,72,76,80]. Similarly, the combination of volumetric parameters on PET with circulating tumor cells (CTC) count and soluble PD-L1 [72,75,83], or lactate dehydrogenase (LDH) [88] has been reported to be as useful for risk stratification. Thanks to the capability of [^18^F]FDG PET/CT to depict underlying immunological status, expressed as bone marrow or lymphatic organ activation (i.e., bone marrow-to-liver ratio, spleen-to-liver ratio) or by the development of irAEs, it is also possible to combine metabolic and immunological parameters to improve response prediction and outcome [48,49,50,51,52,53,56,57,81].

The downside of the previously mentioned findings, despite being fascinating and promising, is that most of the original data derive from retrospective analyses or from limited, single centered, prospective cohorts (Table 2). Consequently, their clinical relevance remains circumscribed to theory, until large prospective multicentric imaging trials are properly conducted.

## 5. Next Generation Imaging for Immunotherapy in Cancer

Radiomics and artificial intelligence (AI) have become a constant mantra in applied sciences, and this includes, necessarily, medical imaging. Automated machine or deep learning algorithms also represent the next frontier of imaging for immunotherapy in cancer, since they might be able to extract precious information, invisible to the naked eye or to conventional measurements. We have known for some years that image heterogeneity is a marker of underlying histological and genetic complexity; but which features could be better associated with specific tumor aspects still requires thorough investigation. What emerges from initial reports published so far on radiomics and AI in the context of immunotherapy setting is that no unique parameter or feature can be defined as superior (Table 2). While features like “skewness” and “kurtosis”, well known from other types of treatment, might represent a marker of treatment failure during ICI in lung cancer [90], for other authors either Small Run Emphasis (SRE), multiparametric radiomics signature (mpRS), cytolytic activity score (CytAct), deeply learned score (DLS), or long zone emphasis (LZE) [89,91,92,93,94] can be as effective. What is missing in this clinical scenario is a solid ground truth, which can only be obtainable from preliminary reports validating imaging parameters with targets specifically relevant for immunotherapy, as in the case of PD-L1 expression. Unfortunately, evidence in this regard is extremely limited, particularly when concerning metabolic imaging [94,101].

**Figure 4:**
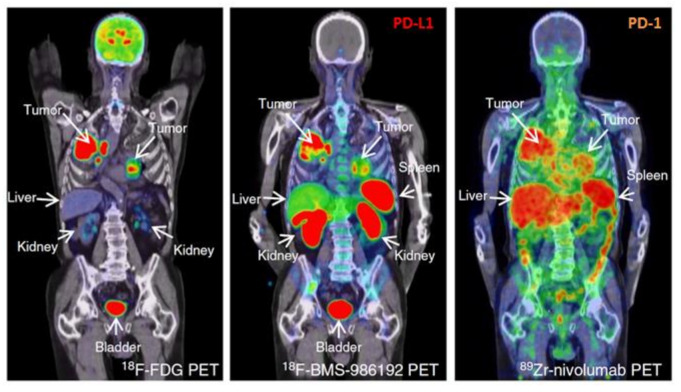
Comparison of [^18^F]FDG PET/CT with anti-PD-L1 (18F-BMS-986192) and anti-PD-1 (89Zr-labeled Nivolumab) immuno-PET images in the same patient with NSCLC. Along the high glucose metabolism of the tumor in both lungs and mediastinal lymph nodes, a heterogeneous tracer uptake for 18F-BMS-986192 PET/CT and 89Zr-labeled Nivolumab PET/CT within and between tumors is demonstrated. Modified from Niemeijer AN et al. Whole body PD-1 and PD-L1 positron emission tomography in patients with non-small-cell lung cancer. Nat Commun 2018;9:4664. [106]; Licensed under a Creative Commons license: http://creativecommons.org/licenses/by/4.0/) Last access date: 2 November 2021.

On the other hand, PET imaging during immunotherapy implies another frontier of development, with radiolabeled immune-based tracers, also known as Immuno-PET. This includes the targeting with radiolabeled antibodies, antibody fragments, or small proteins of checkpoints (i.e., CTLA-4, PD-1, PD-L1) [102,103,104,105,106], tumor infiltrating lymphocytes (ex. CD3, CD4, CD8) [107,108,109,110], cytokines (ex. IL-2) [111], enzymes (ex. Granzyme B, dCK deoxycytidine kinase, dGK deoxyguanosine kinase) [112,113,114,115], and potentially any other element involved in immune system response [116]. The possibility of detecting non-invasively checkpoint expression prior to the administration of ICI, as well as the identification on the entire tumor mass of the amount and pattern of distribution of immune cells, can have priceless clinical implications [106,110]. The same compound used for treatment, ex. ipilimumab, nivolumab, pembrolizumab, atezolizumab, and so forth, [105,106,117,118,119], would be labeled and imaged with PET to detect the actual targeting of tumor sites (Figure 4). Similarly, it would be able to detect the status of lymphocyte activation, exhaustion or cytotoxic capacity by simply injecting radiolabeled molecules targeting enzymes like Granzyme B, a downstream effector of tumoral cytotoxic T cells [113,115,120], or by checking the deoxyribonucleotide kinase activity [112,114]. The majority of data belong mostly to the preclinical setting, with ongoing research aiming to translate the results from bench to clinical practice [106,119,121]. The hope is that in the near future the data will be mature enough to implement immuno-PET into the diagnostic pathway for cancer patient candidates to undergo immunotherapy with checkpoint inhibitors.

## 6. Endnote Remarks

The introduction of immunotherapy in cancer treatment has represented a turning point in medical oncology, but also a new challenge for diagnostic imaging. The multitude of adapted response criteria and the numerous research studies published within a relatively short period of time demonstrate the capability of our community to face challenges and find solutions. From a nuclear medicine point of view, practical directives/guidelines are in the pipeline, along with previously published position papers or comments [122,123] on how to deal with the assessment of tumor response in the era of checkpoint inhibitors. The battlefield should, anyhow, move to clinical validation and recognition by the medical oncology community, which remains skeptical and firmly anchored to morphological criteria. Superior data are required in this regard, since non-inferiority would not be sufficient, given the larger availability of radiological devices (i.e., CT) and the reduced costs of the procedures compared to PET imaging. The astonishing technological leap of the last decade might be the game changer (immune-PET, Radiomics, AI), along with the improved awareness among nuclear medicine physicians of the clinical trial requirements in case of imaging studies, which should represent the backbone of any novel clinical indication or new tracer development.

## Figures and Tables

**Table 1:** Summary of anatomic and metabolic criteria for immunotherapy response assessment.

Criteria					
Morphologic	CR	PR	SD	PD	New Lesions
**RECIST 1.1 (2009)** [6]	disappearance of all lesions	≥30% decrease from baseline	Neither PR nor PD	≥20% increase, minimum 5 mm	as progressive disease
**irRC (2009)** [7]	as RECIST 1.1	≥50% decrease from baseline	<50% decrease in tumor burden vs. baseline or <25% increase vs. nadir	≥25% increase	incorporated into tumor burden; confirmed at least 4 weeks apart
**irRECIST (2013)** [8]	as RECIST 1.1	as RECIST 1.1	Neither PR nor PD	as RECIST 1.1	same as irRC
**iRECIST (2017)** [9]	as RECIST 1.1	as RECIST 1.1	Neither PR nor PD	as RECIST 1.1	iUPD, not incorporated into tumor burden; confirmed 4–12 weeks apart (iCPD)
**imRECIST (2018)** [10]	as RECIST 1.1	as RECIST 1.1	Neither PR nor PD	as RECIST 1.1	same as irRC
**Metabolic**	**CMR**	**PMR**	**SMD**	**PMD**	**New lesions**
**EORTC (1999)** [11]	complete resolution of [^18^F]FDG uptake	reduction of a minimum of 15% ± 25% in tumor SUV after 1 cycle of chemotherapy, and >25% after more than one treatment cycle	increase in SUV of less than 25% or a decrease of less than 15%	increase in tumor FDG uptake > 25%, increase of the maximum tumor > 20%, new metastases	as progressive disease
**PERCIST (2009)** [12]	disappearance of all metabolically active lesions	SULpeak reduction ≥ 30% in the hottest target lesions	neither PMD nor PMR/CMR	SULpeak increase ≥ 30% in the hottest target lesion	as progressive disease
**PERCIMT (2018)** [13]	disappearance of all metabolically active lesions	disappearance of some but not all metabolic lesions and no new lesions	neither PMD nor PMR/CMR	4 or more new lesions (<1 cm in diameter), or 3 or more new lesions (>1 cm in diameter), or 2 or more new lesions (>1.5 cm in diameter)	according to the number and the diameter
**imPERCIST (2019)** [14]	same as PERCIST	same as PERCIST	neither PMD nor PMR/CMR	SULpeak increase ≥ 30% in the hottest target lesion	do not configure automatically PMD, incorporate in the sum of SULpeak
**iPERCIST (2019)** [15]	same as PERCIST	same as PERCIST	neither PMD nor PMR/CMR	SULpeak increase ≥ 30%, or new [^18^F]FDG-avid lesions (UPMD)	need to be confirmed after 4–8 weeks (CPMD)
**Combined criteria**	**Clinical benefit**			**No clinical benefit**	
**PECRIT (2017)** [16]	CR as per RECIST 1.1 (disappearance of all target lesions; reduction in short axis of target lymph nodes to <1 cm; no new lesions)	PR as per RECIST 1.1 (decrease in target lesion diameter sum > 30%)	Does not meet other criteria plus change in SUL peak of the hottest lesion of >15%	Does not meet other criteria plus change in SUL peak of the hottest lesionof ≤15%	PD as per RECIST 1.1 (increase in target lesion diameter sum of >20% and at least 5 mm or new lesions)

**Table 2:** Summary of major studies investigating PET/CT for immunotherapy response assessment and outcome prediction.

Author	Year	Study	Histology	Number	Treatment	Used Criteria	Key Message	Reference
Summary of Studies Investigating Melanoma								
Kong et al.	2016	prospective	melanoma	27	pembrolizumab, nivolumab	irRC, Deauville criteria, SUVmax	Residual metastases after a prolonged period without progression on anti-PD-1 therapy may be metabolically inactive	[44]
Cho et al.	2017	prospective	melanoma	20	ipilimumab nivolumab	PECRIT	Combined metabolic and anatomic parameters predict response with 95% accuracy	[16]
Seith et al.	2018	retrospective	melanoma	10	ipilimumab	PERCIST	Complete responders identified as early 2 weeks	[45]
Anwar et al.	2018	prospective	melanoma	41	ipilimumab	PERCIMT	A threshold of 4 new [^18^F]FDG-avid lesions led to a sensitivity (correctly predicting CB) of 84% and a specificity (correctly predicting No-CB) of 100%	[13]
Tan et al.	2018	retrospective	melanoma	104	anti-PD-1 or plus ipilimumab	RECIST, EORTC	RECIST PFS post 1-year landmark was similar in patients with CR versus PR/SD, but improved in patients with CMR versus non-CMR. Also PFS in patients with PR on CT improved.	[46]
Sachpekidis et al.	2018	prospective	melanoma	41	ipilimumab	EORCT, PERCIMT	PERCIMT had a significantly higher sensitivity than EORTC (*p* = 0.004), while there was no significant difference in specificity (*p* = 0.5).	[38]
Amrane et al.	2019	retrospective	melanoma	37	ipilimumab plus pembrolizumab, nivolumab	RECIST1.1 iRECIST PERCIST PECRIT	RECIST1.1, iRECIST, and PERCIST were predictive for PFS and OS	[39]
Ito et al.	2019	retrospective	melanoma	60	ipilimumab	imPERCIST, PERCIST1, PERCIST5	imPERCIST5 responders had a longer 2-y OS, 66% versus 29% for vs. nonresponders (*p* = 0.003). imPERCIST remained prognostic at multivariate analysis	[14]
Ito et al.	2019	retrospective	melanoma	142	ipilimumab	MTV	Baseline MTV as prognostic factor	[47]
Boursi et al.	2019	retrospective	melanoma	14	ipilimumab	colonic SUV	Colonic SUVmax higher for complete responders	[48]
Sachpekidis et al.	2019	retrospective	melanoma	41	ipilimumab	lymphoid organs metabolism	The appearance of sarcoid-like lymphadenopathy correlated to clinical benefit of anti-CTLA-4 therapy	[49]
Sachpekidis et al.	2019	retrospective	melanoma	16	vemurafenib plus ipilimumab	EORTC, PERCIMT	PERCIMT criteria correctly classified more patients than EORTC criteria. Radiologic signs of irAEs, such as colitis and arthritis, predicted significantly longer PFS than those without irAEs (*p* = 0.036)	[50]
Seban et al.	2019	retrospective	melanoma	55	anti-PD-1	RECIST1.1, TMTV, TLG, BLR, SLR	Low TMTV and TLG correlated with BOR, while hematopoietic tissue metabolism, i.e., BLR (Bone marrow-to-Liver SUVmax ratio), and SLR (Spleen-to-Liver SUVmax ratio), correlates inversely with survival.	[51]
Nobashi et al.	2019	retrospective	melanoma, lymphoma, renal cell carcinoma	40	ipilimumab nivolumab, pembrolizumab	SUVs in tumor and lymphoid organs	PET-detectable irAEs were predictive of a favorable outcome. In particular, early development of thyroiditis.	[52]
Seban et al.	2020	retrospective	mucosal melanoma (Muc-M) or cutaneous melanoma (Cut-M)	56	ipilimumab pembrolizumab	RECIST1.1, SUVmax, TMTV, TLG, BLR	For Muc-M, high baseline SUVmax was associated with shorter OS, whereas for Cut-M, baseline increased TMTV and increased BLR were associated with shorter OS, shorter PFS, and lower response (ORR, DCR)	[53]
Iravani et al.	2020	retrospective	melanoma	31	nivolumab plus ipilimumab	PERCIST, wbMTV (whole body MTV)	Patients with PMD had significantly higher pre-treatment wbMTV.	[54]
Nakamoto et al.	2020	retrospective	melanoma	85	nivolumab, ipilimumab pembrolizumab	MTV	MTVpost and the presence of central nervous system lesions were independent prognostic factors for OS.	[55]
Wong et al.	2020	retrospective	melanoma	90	anti-PD-1 and or ipilimumab	SUVmax, MTV, and spleen to liver ratio (SLR)	SLR was associated with poor OS in a multi-variable model independent of stage, LDH, absolute lymphocyte count and MTV.	[56]
Seith et al.	2020	prospective	melanoma	17	anti-CTLA-4 and/or anti-PD-1	iRECIST, PERCIST, ADC, SULmean spleen, SULmean bone marrow	Responder group presents with an increased spleen volume and metabolic activity of bone marrow.	[57]
Annovazzi et al.	2020	retrospective	melanoma	57	Ipilimumab nivolumab, pembrolizumab	RECIST 1.1, EORTC, PERCIMT, MTV, TLG (up to 5 target lesions)	Best predictor of therapy response was MTV combined with PERCIMT for ipilimumab; for anti-PD-1 therapy EORTC, MTV, and TLG.	[58]
Nakamoto et al.	2020	retrospective	melanoma	76	ipilimumab nivolumab, pembrolizumab, nivolumab plus ipilimumab	irRECIST, MTV, total measured tumor burden (TMTB)	MTVbase of HPD patients was larger than that of non-HPD. HPD patients demonstrated shorter median OS	[59]
Prigent et al.	2021	retrospective	melanoma	29	nivolumab, pembrolizumab, nivolumab plus ipilimumab	imPERCIST5, whole-body metabolic active tumor volume (WB-MATV), bone-to-liver (BLR), SLR	Mean spleen-to-liver (SLRmean) increase greater than 25% at 3 months, compared with baseline, was associated with poor outcome	[60]
Sachpekidis et al.	2021	retrospective	melanoma	31	ipilimumab, pembrolizumab, nivolumab plus ipilimumab	EORTC, PERCIMT, SLRmean, SLRmax	PET/CT, performed after two ICIs’ cycles, can identify the majority of non-responders	[61]
Sachpekidis et al.	2021	prospective	melanoma	25	nivolumab, pembrolizumab, nivolumab plus ipilimumab	SUVmean, SUVmax and quantitative on dynamic PET (K1, k3, influx, FD, fractal dimension)	SUVmean, SUVmax and FD adversely affected PFS	[62]
Schank et al.	2021	retrospective	melanoma	45	nivolumab, pembrolizumab, ipilimumab, nivolumab plus ipilimumab	EORTC, PERCIMT	Two-year PFS was 94% among CMR patients and 62% among non-CMR patients	[63]
Nakamoto et al.	2021	retrospective	melanoma	92	pembrolizumab, nivolumab, nivolumab and ipilimumab, nivolumab and relatlimab (anti-LAG-3 antibody)	iRECIST, SUVmax, MTV, BLR	High BLR were associated with poor PFS and OS	[64]
Kitajima et al.	2021	retrospective	melanoma	27	Nivolumab, pembrolizumab	EORTC, PERCIST, and imPERCIST	Responders (CMR/PMR) showed significantly longer PFS and OS than non-responders (SMD/PMD)	[65]
**Summary of studies investigating lung cancer**								
Grizzi et al.	2018	prospective	NSCLC	17	nivolumab, pembrolizumab	SUVmax, SUVmean	Antithetical correlation between baseline parameters and response	[66]
Kaira et al.	2018	prospective	NSCLC	24	nivolumab	SUVmax, MTV, TLG	TLG at 1 months was predictive for worse PFS and OS	[67]
Jreige et al.	2019	retrospective	NSCLC	49	pembrolizumab, nivolumab, durvalumab, atezolizumab	SUVmax, SUVmean, MTV, TLG, MMVR	MMVR (metabolic-to-morphological volume ratio) was predictive for clinical benefit	[68]
Goldfard et al.	2019	retrospective	NSCLC	28	nivolumab	iRECIST, iPERCIST	In comparison with iRECIST, iPERCIST showed reclassification in 39% of patients.	[15]
Rossi et al.	2019	prospective	NSCLC	72	nivolumab	RECIST1.1 irRC PERCIST imPERCIST	Added prognostic value for PERCIST imPERCIST in patients with PD according irRC	[41]
Evangelista et al.	2019	retrospective	NSCLC	32	nivolumab	SUVmax, MTV, TLG	SUVmax higher in non-responders women than men	[69]
Takada et al.	2019	retrospective	NSCLC	89	nivolumab, pembrolizumab	RECIST 1.1 SUVmax	The response rate of patients with SUVmax value ≥ 11.16 (41.3%) was significantly higher than that of patients with SUVmax < 11.16 (11.6%, *p* = 0.0012)	[70]
Beer et al.	2019	prospective	NSCLC	42	nivolumab, pembrolizumab, durvalumab	RECIST 1.1, iRECIST, and PERCIST	There was only a slight agreement between RECIST 1.1 and PERCIST 1.0 and PERCIST 1.0 and iRECIST. Median PFS and OS, as were significantly longer for responders for all criteria, with no significant difference between them.	[40]
Seban et al.	2020	retrospective	NSCLC	80	nivolumab, pembrolizumab, atezolizumab	RECIST1.1, TMTV	Baseline TMTV and dNLR were associated with poor OS and absence of DCB (disease clinical benefit)	[71]
Humbert et al.	2020	prospective	NSCLC	50	nivolumab, ipilimumab	PERCIST	Pseudoprogression and iDR (immune dissociated-response) associated with clinical benefit	[30]
Castello et al.	2020	prospective	NSCLC	46	nivolumab, ipilimumab pembrolizumab	SUVmax, SUVmean, MTV, TLG	Baseline MTV and dNLR predictors for hyperprogression	[28]
Castello et al.	2020	prospective	NSCLC	35	nivolumab, nivolumab plus ipilumimab pembrolizumab	RECIST 1.1, EORTC, SUVmax, MTV, TLG	CTC count variation (ΔCTC) was significantly associated with tumor metabolic response. CTC count at 8 weeks was an independent predictor for PFS and OS, whereas ΔMTV and ΔSUVmax were predictive for PFS and OS, respectively.	[72]
Seban et al.	2020	retrospective	NSCLC	63	pembrolizumab	RECIST1.1, TMTV	Metabolic score combining TMTV on the baseline and pretreatment dNLR (derived neutrophils-to-lymphocytes ratio) was associated with the survival and response	[73]
Chardin et al.	2020	prospective	NSCLC	75	nivolumab, pembrolizumab	SUVmax, SUVpeak, MTV and TLG	A high MTV and TLG were significantly associated with a lower OS. MTV and TLG could reliably predict ETD (early treatment discontinuation)	[74]
Castello et al.	2020	prospective	NSCLC	20	nivolumab, nivolumab plus ipilumimab pembrolizumab	iRECIST, imPERCIST	Association of elevated sPD-L1 (soluble PD-L1), and high MTV.	[75]
Castello et al.	2020	prospective	NSCLC	35	nivolumab, pembrolizumab	RECIST 1.1, imRECIST, EORTC, PERCIST, imPERCIST, and PERCIMT	Low agreement between imRECIST and imPERCIST. Performance status, imRECIST and imPERCIST were predictive for PFS, while only performance status and imPERCIST were predictive for OS	[43]
Castello et al.	2020	prospective	NSCLC	33	nivolumab, pembrolizumab	iRECIST, EORTC, SUVmax, SUVmean, MTV, TLG	An immune-metabolic-prognostic index (IMPI), based on post-NLR and post-TLG was developed, resulting predictive for both PFS and OS.	[76]
Tao et al.	2020	prospective	NSCLC	36	neoadjuvant sintilimab	PERCIST, SULmax, SULpeak, MTV, TLG, ΔSULmax%, ΔSULpeak%, ΔMTV%, ΔTLG%	All PMR tumors showed MPR (major pathologic response). The degree of pathological regression was positively correlated with SULmax of scan-1, and negatively correlated with all metabolic parameters of scan-2.	[77]
Hashimoto et al.	2020	retrospective	NSCLC	85	nivolumab, pembrolizumab	RECIST1.1, SUVmax, SUVmean, MTV, TLG	TLG and MTV are independent prognostic factors for outcome after anti-PD-1 antibody.	[78]
Umeda et al.	2020	prospective	NSCLC	25	nivolumab	RECIST1.1, ΔTLG, ΔADCmean	A cut-off of 16.5 for ΔTLG + ΔADCmean had the highest accuracy (92%) for distinguishing PD, and was an independent predictor of shorter PFS and OS.	[79]
Seban et al.	2020	retrospective	NSCLC	63	upfront pembrolizumab	SUVmax, SUVmean, TMTV and TLG	Baseline low TMTV and high tumor SUVmean correlate with survival and LTB (long-term benefit)	[80]
Cvetkovic et al.	2021	retrospective	NSCLC	71	anti-PD-1/PD-L1 monotherapy or in combination with chemotherapy	average colon SUVmax	Lower colon physiologic [^18^F]FDG uptake prior to ICI was associated with better clinical outcomes and higher gut microbiome diversity	[81]
Ito et al.	2021	retrospective	NSCLC	58	PD-1 or PD-L1 inhibitor therapy	EORTC5, PERCIST5, imPERCIST5	After SUV harmonization with dedicated software packages “RAVAT” and “RC Tool for Harmonization, response criteria was associated with OS.	[82]
Bauckneht et al.	2021	prospective	NSCLC	45	nivolumab, pembrolizumab	RECIST 1.1, NLR, dNLR, lymphocyte-to-monocyte ratio (LMR), platelets-to-lymphocyte ratio (PLR), systemic inflammation index (SII), SUVmax, MTV, TLG	The combined parameters into the IMPI (immune metabolic prognostic index) significantly differentiated OS in NSCLC (*p* < 0.0001)	[83]
Ferdinandus et al.	2021	retrospective	NSCLC	45	Atezolizumab, Nivolumab, pembrolizumab, ipilimumab/nivolumab	RECIST 1.1, background level (using mediastinum as reference) for CMR.	CMR after 24 months allows for a safe discontinuation of ICI	[84]
Castello et al.	2021	prospective	NSCLC	50	nivolumab, pembrolizumab, atezolizumab	iRECIST, EORTC, MTV, TLG and their variations	ATB therapy is associated with a worse response, PFS, and higher metabolic tumor burden in NSCLC	[85]
Ayati et al.	2021	retrospective	NSCLC	72	nivolumab, pembrolizumab	RECIST, iRECIST, PERCIST, imPERCIST	Most FDG-avid lesions based on PERCIST and imPERCIST reflect the overall metabolic response	[42]
Vekens et al.	2021	retrospective	NSCLC	30	pembrolizumab	RECIST 1.1, SUVmax, TMTV, TLG	TMTV and TLG were associated with PFS and OS, while RECIST 1.1 were not	[86]
Park et al.	2021	retrospective	NSCLC	24	nivolumab, pembrolizumab	EORCT, PERCIST, RECIST 1.1	metabolic parameters were independent factors for predicting progression	[87]
Ke et al.	2021	retrospective	Lung cancer (SCLC; NSCLC)	120	PD-1/PD-L1 blockade plus chemotherapy	iRECIST, SUVmax, SUVmean, SUVpeak, MTV, TLG, lactate dehydrogenase (LDH), dNLR	The combination of SUVmax plus LDH was an independent predictor of OS	[88]
**Summary of studies investigating Radiomics and AI**								
Valentinuzzi et al.	2020	prospective	NSCLC	30	pembrolizumab	iRECIST, iRADIOMICS	Multivariate iRADIOMICS, in particular Small Run Emphasis (SRE), showed a more predictive power compared to PD-L1 and iRECIST.	[89]
Polverani et al.	2020	Retrospective	NSCLC	57	anti-PD-1 or anti-PD-L1	RECIST1.1, SUVmax, MTV, TLG, radiomics feature	Patients with high MTV, TLG and heterogeneity expressed by “skewness” and “kurtosis” had a higher probability of failing immunotherapy.	[90]
Mu et al.	2020	Retrospective/prospective	NSCLC	99 and 48	anti-PD-L1	RECIST1.1, mpRS (multiparametric radiomics signature)	mpRS could predict patients who will receive DCB (durable clinical benefit)	[91]
Park et al.	2020	Retrospective	Lung adenocarcinoma	59	immune checkpoint blockade in monotherapy	RECIST 1.1, cytolytic activity score (CytAct)	Higher minimum predicted CytAct in associated with significantly prolonged PFS and OS	[92]
Flaus et al.	2021	retrospective	melanoma	56	Nivolumab or Pembrolizumab	MTV and forty-one IBSI compliant parameters	MTV and long zone emphasis (LZE) correlated with shorter OS and defined three risk categories for the prognostic score	[93]
Mu et al.	2021	Retrospective/prospective	NSCLC	697	ICI	RECIST 1.1, deeply learned score (DLS)	PD-L1 DLS significantly discriminated PD-L1 positive and negative patients; combining DLS with clinical characteristics accurately predicts DCB, PFS, and OS	[94]

Notes: PubMed database was searched from 2010 until September 2021 for the terms: (“fluorodeoxyglucose f18” OR (“fluorodeoxyglucose” AND “f18”) OR “fluorodeoxyglucose f18” OR (“18f”AND “fdg”) OR “18f fdg”) AND “pet” AND (“immunotherapy” OR “immunotherapies” OR “immunotherapy s”) AND (“cancer s” OR “cancerated” OR “canceration” OR “cancerization” OR “cancerized” OR “cancerous” OR “neoplasms”OR “cancer” OR “cancers”).

## Data Availability

The data presented in this study are available on motivated request to the corresponding author.

## Abbreviations

PERCIST	PET Response Criteria in Solid Tumors
PECRIT	PET/CT Criteria for Early Prediction of Response to Immune Checkpoint Inhibitor Therapy (combined RECIST 1.1 and PERCIST)
PERCIMT	PET Response Evaluation Criteria for Immunotherapy
CMR	complete metabolic response
PMR	partial metabolic response
SMD	stable metabolic disease
PMD	progressive metabolic disease
SULpeak	lean body mass corrected SUV peak
UPMD	unconfirmed progressive metabolic disease
CPMD	confirmed progressive metabolic disease.
RECIST	Response Evaluation Criteria in Solid Tumors
irRC	immune-related Response Criteria
CR	complete response
PR	partial response
SD	stable disease
PD	progressive disease
iUPD	initially unconfirmed progressive disease
iCPD	confirmed progressive disease
CB	clinical benefit
EORTC	European Organization for Research and Treatment of Cancer (EORTC5, includes the sum of SUVmax)
MTV	metabolic tumor volume
wbMTV	whole body MTV
TMTV	total metabolic tumor volume
WB-MATV	whole body metabolically active tumor volume
TLG	total lesions glycolysis
iDR	immune dissociated-response
ETD	early treatment discontinuation
BLR	bone marrow-to-liver SUVmax ratio
SLR	spleen-to-liver SUVmax ratio
dNLR	derived neutrophils-to-lymphocytes ratio
LDH	lactate dehydrogenase
FD	fractal dimension
ICI	immune checkpoint inhibitors
irAEs	immune-related adverse events
IMPI	immune-metabolic-prognostic index
ATB	antibiotic
ADC	apparent diffusion coefficient
SRE	Small Run Emphasis
mpRS	multiparametric radiomics signature
DLS	deeply learned score
DCB	durable clinical benefit
PFS	progression-free survival
OS	overall survival
DCR	disease control rate
ORR	overall response rate
Muc-M	mucosal melanoma
Cut-M	cutaneous melanoma
sPD-L1	soluble PD-L1.
